# Efficacy and safety of autologous peripheral blood stem cell transplantation for Philadelphia chromosome-positive acute lymphoblastic leukemia: A study protocol for a multicenter exploratory prospective study (Auto-Ph17 study)

**DOI:** 10.1097/MD.0000000000009568

**Published:** 2017-12-29

**Authors:** Satoshi Nishiwaki, Isamu Sugiura, Yasuhiko Miyata, Shigeki Saito, Masashi Sawa, Tetsuya Nishida, Koichi Miyamura, Yachiyo Kuwatsuka, Akio Kohno, Masaaki Yuge, Masanobu Kasai, Hiroatsu Iida, Shingo Kurahashi, Masahide Osaki, Tatsunori Goto, Seitaro Terakura, Makoto Murata, Hiroyoshi Nishikawa, Hitoshi Kiyoi

**Affiliations:** a Center for Advanced Medicine and Clinical Research, Nagoya University Hospital, Nagoya; b Division of Hematology and Oncology, Toyohashi Municipal Hospital, Toyohashi; c Department of Hematology, National Hospital Organization Nagoya Medical Center; d Department of Hematology and Oncology, Japanese Red Cross Nagoya Daini Hospital, Nagoya; e Department of Hematology, Anjo Kosei Hospital, Anjo; f Department of Hematology and Oncology, Nagoya University Graduate School of Medicine; g Department of Hematology, Japanese Red Cross Nagoya Daiichi Hospital, Nagoya; h Department of Hematology and Oncology, JA Aichi Konan Kosei Hospital, Konan; i Division of Hematology, Ichinomiya Municipal Hospital, Ichinomiya; j Department of Immunology, Nagoya University Graduate School of Medicine, Nagoya, Japan.

**Keywords:** autologous peripheral blood stem cell transplantation, dasatinib, efficacy, Philadelphia chromosome positive acute lymphoblastic leukemia, safety

## Abstract

**Introduction:**

The prognosis of Philadelphia chromosome positive acute lymphoblastic leukemia (Ph + ALL) has been dramatically improved since the introduction of tyrosine kinase inhibitors (TKIs). Although allogeneic hematopoietic cell transplantation (allo-HCT) is a major treatment option, the role of autologous peripheral blood stem cell transplantation (auto-PBSCT) has been reconsidered, especially in patients who achieved early molecular remission.

**Methods and analysis:**

This is a multicenter exploratory study for Ph + ALL patients aged between 55 and 70 years who achieved complete molecular remission within 3 cycles of chemotherapy. The target sample size is 5, and the registration period is 2 years. The primary endpoint is Day100- mortality after transplantation, and the secondary endpoints are survival, relapse rate, nonrelapse mortality, and adverse events.

This study is divided into 3 phases: peripheral blood stem cell harvest, transplantation, and maintenance. Chemomobilization is performed using a combination of cyclophosphamide (CPM), doxorubicin, vincristine (VCR), and prednisolone (PSL). As a preparative regimen, the LEED regimen is used, which consists of melphalan, CPM, etoposide, and dexamethasone. Twelve cycles of maintenance therapy using a combination of VCR, PSL, and dasatinib are performed.

In association with relapse, the minimal residual disease (MRD) of *BCR-ABL* chimeric gene and T-cell subsets are analyzed both before and after auto-PBSCT.

**Ethics and dissemination:**

The protocol was approved by the institutional review board of Nagoya University Hospital and all the participating hospitals. Written informed consent was obtained from all patients before registration, in accordance with the Declaration of Helsinki. Results of the study will be disseminated via publications in peer-reviewed journals.

**Trial registration:**

Trial registration number UMIN000026445.

## 1 Introduction

The role of autologous peripheral blood stem cell transplantation (auto-PBSCT) for Philadelphia chromosome positive acute lymphoblastic leukemia (Ph + ALL) has changed in the era of tyrosine kinase inhibitors (TKIs), although allogeneic hematopoietic cell transplantation (allo-HCT) is still considered to be an option to cure Ph + ALL.^[1–5]^ In the Cancer and Leukemia Group B (CALGB) 10001 study, overall survival (OS) (median 6.0 years vs not reached) and disease-free survival (DFS) (median 3.5 vs 4.1 years) were similar between patients with a partial or complete molecular response who had undergone autologous transplantation and those who had undergone allo-HCT.^[6]^ In addition, in patients achieving a major molecular response, the outcome was similar between patients who had undergone autologous transplantation and those who had undergone allo-HCT [OS: hazard ratio (HR) 0.94, 95% confidence interval (95% CI) 0.53–1.65, *P* = .82; DFS: HR 0.95, 95% CI 0.51–1.74, *P* = .95] in the study of the Group for Research on Adult Acute Lymphoblastic Leukemia (GRAALL).^[7]^ Because hematological complete remission (CR) has been achieved in many patients recently,^[8,9]^ monitoring of minimal residual disease (MRD) is important for long-term disease control.^[10–15]^

Age is one of the most important prognostic factors in patients who underwent allo-HCT, although the outcome of allo-HCT is generally favorable in Japan (http://www.jdchct.or.jp/en/data/slide/2016/). In Ph + ALL patients, an age of 55 years or older has been identified as a risk factor for nonrelapse mortality (NRM) after allo-HCT.^[15]^ On the contrary, the risk of NRM is much lower in auto-PBSCT, and auto-PBSCT has been performed on patients around 70 and up to 75 years old.^[16]^

In this study, we planned to analyze the safety and efficacy of auto-PBSCT for Ph + ALL patients aged between 55 and 70 years with an early molecular response. In addition, immune recovery after auto-PBSCT is also a subject of interest, especially the function of T cells.

## 2 Objectives

### 2.1 Primary

The primary endpoint is Day 100- mortality after transplantation.

### 2.2 Secondary

The secondary endpoints are as follows:

(1) Day100- molecular and hematological relapse rate;
(2) 1-year molecular and hematological relapse rate;
(3) 3-year molecular and hematological relapse rate;
(4) Day100- OS, DFS, relapse rate, and NRM;
(5) 1-year OS, DFS, relapse rate, and NRM;
(6) 3-year OS, DFS, relapse rate, and NRM;
(7) The proportion of therapy-related mortality;
(8) The proportion of adverse events in each regimen;
(9) Success rate of PBSCH;
(10) Detection of *BCR-ABL* chimeric gene in harvested peripheral blood stem cells by real-time quantitative polymerase chain reaction (RQ-PCR);
(11) Safety of PBSCT (the proportion of engraftment and engraftment failure);
(12) Cumulative dose of dasatinib (DA) during maintenance therapy;
(13) Mutation analysis of the *BCR-ABL* chimeric gene in relapsed patients.

## 3 Methods and analysis

### 3.1 Study design

**Figure 1:**
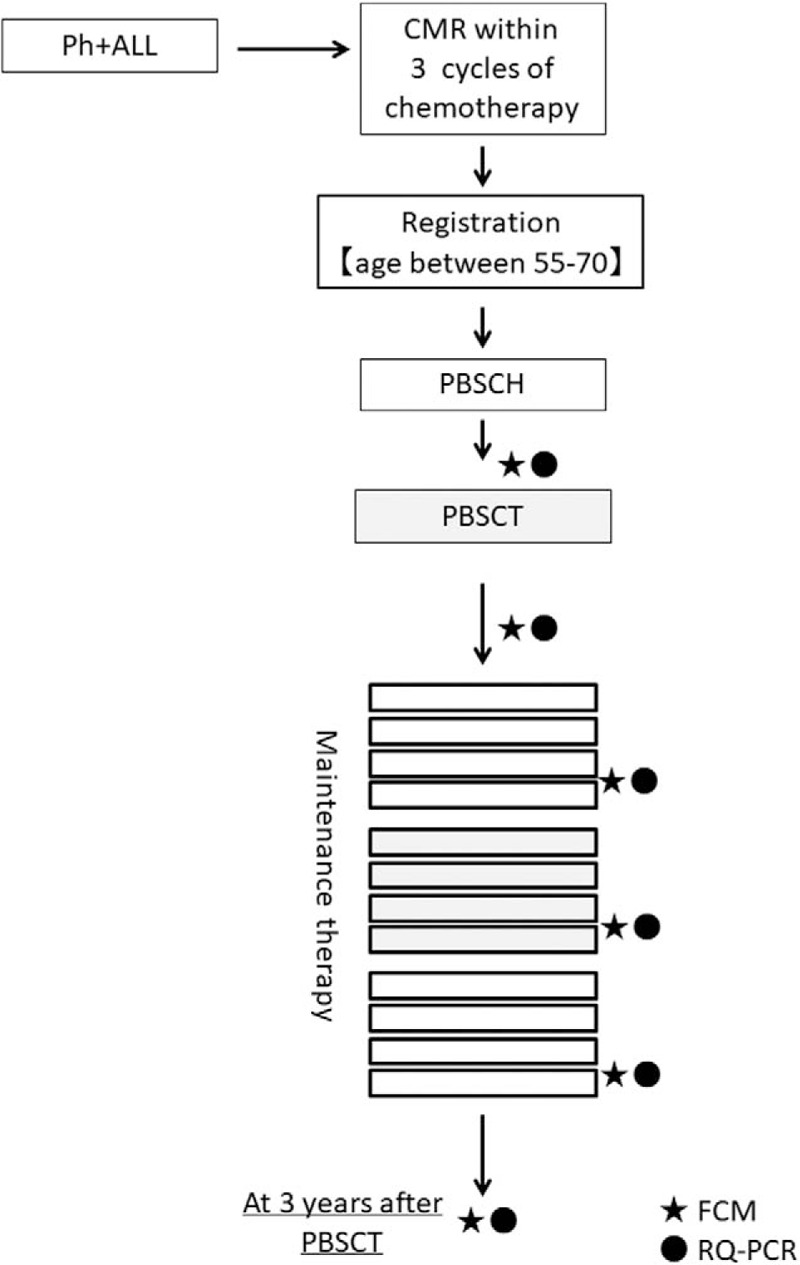
Outline of the Auto-Ph17 study. CMR = complete molecular remission, FCM = flow cytometry, PBSCH = peripheral blood stem cell harvest, PBSCT = peripheral blood stem cell transplantation, Ph + ALL = Philadelphia chromosome-positive acute lymphoblastic leukemia, RQ-PCR = real-time quantitative polymerase chain reaction.

This is a multicenter exploratory study of auto-PBSCT for Ph + ALL. This study is divided into 3 phases: peripheral blood stem cell harvest (PBSCH), transplantation, and maintenance (Fig. 1). Because this is an exploratory study, the target sample size is 5, and the registration period is 2 years. This study was registered in the UMIN Clinical Trials Registry with the identifier UMIN000026445.

### 3.2 Study setting

Eight hospitals in Aichi Prefecture agreed to take part in this study: Anjo Kosei Hospital, Ichinomiya Municipal Hospital, Konan Kosei Hospital, Toyohashi Municipal Hospital, Nagoya Medical Center, Japanese Red Cross Nagoya Daiichi Hospital, Japanese Red Cross Nagoya Daini Hospital, and Nagoya University Hospital. The protocol was approved by the institutional review board of each hospital (the latest edition ver. 1.1 13/Jun/2017). Written informed consent was obtained from all patients before registration, in accordance with the Declaration of Helsinki.

Patients are registered in this study after the independent review by the Data center in the Center for Advanced Medicine and Clinical Research of Nagoya University Hospital, where the inclusion and exclusion criteria are checked. Independent monitoring will be planned at least annually according to the Japanese clinical trial guideline.

### 3.3 Participants

The inclusion criteria are as follows:

(1) Acute lymphoblastic leukemia [B-lymphoblastic leukemia/lymphoma of WHO classification (5th edition)].
(2) BCR/ABL positive.
(3) Patients aged between 55 and 70 years.
(4) Newly diagnosed patients.
(5) Complete molecular remission (CMR) within 3 chemotherapy regimens.
(6) The Eastern Cooperative Oncology Group (ECOG) performance status of 0, 1, 2, or 3.
(7) Adequate function of key organs:
  a) Cardiac; No serious abnormal findings on electrocardiogram or echocardiogram.
  b) Hepatic; Serum total bilirubin≦2.0 mg/dL.
  c) Renal; Serum creatinine≦2.0 mg/dL.
  d) Pulmonary; Percutaneous oxygen saturation≧94%
(8) Voluntary written consent is given before enrollment.

CMR is defined by the absence of detectable MRD with a sensitivity of at least 0.01%.^[7]^

Exclusion criteria are as follows:

1. Heart insufficiency:
  1) Uncontrolled angina or heart failure, or myocardial infarction within 3 months.
  2) Congenital long QT syndrome.
  3) Ventricular arrhythmia (ventricular tachycardia, ventricular fibrillation, Torsades de pointes),
  4) QTc≧481 ms
2. Pulmonary fibrosis, interstitial pneumonitis.
3. Uncontrollable diabetes mellitus:
  1) Fasting blood sugar > 250 mg/dL even with insulin administration.
  2) Hypoglycemic attack twice or more/day due to insulin administration
4. Grade 4 infection.
5. HIV antibody positive.
6. HBs antigen positive.
7. Acquired bleeding diathesis.
8. Psychiatric illness.
9. Active another malignancy.
10. Patients who, in the judgment of the investigator, are inappropriate for entry into this study.

### 3.4 Study procedures-PBSCH

**Figure 2:**
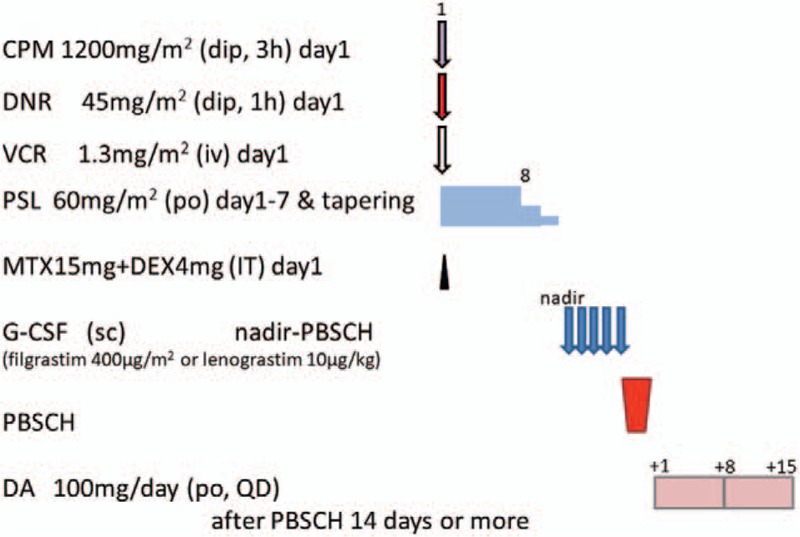
Chemomobilization regimen for peripheral blood stem cell harvest. CPM = cyclophosphamide, DA = dasatinib, DNR = doxorubicin, G-CSF = Granulocyte colony-stimulating factor, IT = intrathecal injection, MTX = methotrexate, PBSCH = peripheral blood stem cell harvest, PSL = prednisolone, VCR = vincristine.

Chemomobilization is performed for PBSCH: cyclophosphamide (CPM) 1200 mg/m^2^ (65 years≧; 750 mg/m^2^) on Day 1, doxorubicin 45 mg/m^2^ (65 years≧; 40 mg/m^2^) on Day 1, vincristine (VCR) 1.3 mg/m^2^ (65 years≧; 1 mg/m^2^) on Day 1, prednisolone (PSL) 60 mg/m^2^ (65 years≧; 40 mg/m^2^) on Days 1 to 7, and intrathecal injection of methotrexate 15 mg and dexamethasone (DEX) 4 mg on Day 1 (Fig. 2). Granulocyte colony-stimulating factor (G-CSF) (filgrastim 400 μg/m^2^ or lenograstim 10 μg/kg s.c.) is initiated in the neutropenic phase and continued until the end of PBSCH. PBSCH is initiated on the day when the WBC count is around 5 x 10^9^/L. PBSCH is finished when 2 x 10^6^/kg or more CD34+ cells are collected. The second PBSCH is performed when the total corrected CD34+ cells were less than 2 x 10^6^/kg after 3 days of PBSCH.

The second PBSCH is performed using the CHASE regimen, which is used for PBSCH in lymphoma patients^[17,18]^: CPM 1200 mg/m^2^ (65 years≧; 750 mg/m^2^) on Day1, cytarabine 2 g/m^2^ (60 years≧; 1 g/m^2^, 65 years≧; 500 mg/m^2^) on Days 2 and 3, etoposide (ETP) 100 mg/m^2^ on Days 1 to 3, and DEX 40 mg/body (65 years≧; 20 mg/body) on Days 1 to 3.

DA 100 mg/day is administered for 14 days or longer from the day following the date of last PBSCH.

### 3.5 Study procedures-transplantation

**Figure 3:**
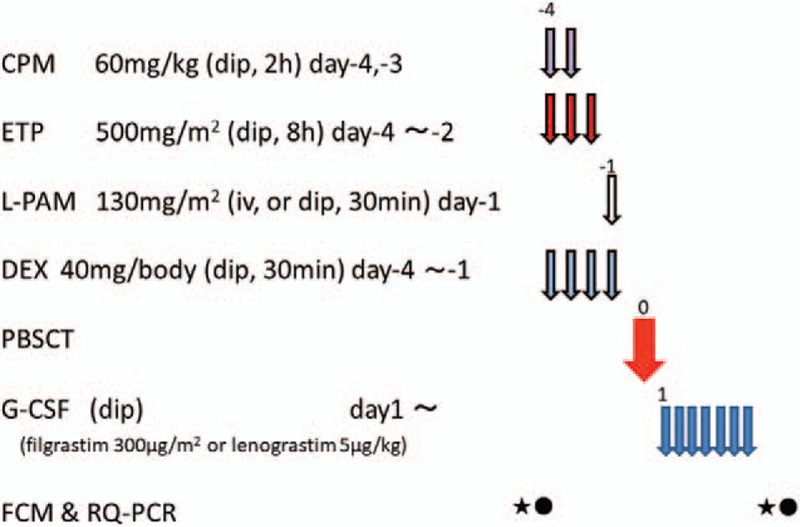
Preparative regimen for peripheral blood stem cell transplantation (the LEED regimen). CPM = cyclophosphamide, DEX = dexamethasone, ETP = etoposide, FCM = flow cytometry, G-CSF = Granulocyte colony-stimulating factor, L-PAM = melphalan, PBSCT = peripheral blood stem cell transplantation, RQ-PCR = real-time quantitative polymerase chain reaction.

The LEED regimen is used as a preparative regimen for autologous PBSCT, which is used for autologous PBSCT in lymphoma patients^[17,19]^: melphalan 130 mg/m^2^ on Day–1, CPM 60 mg/kg on Days –4 and –3, ETP 500 mg/m^2^ on Days –4 to –2, and DEX 40 mg/body on Days –4 to –1 (Fig. 3). G-CSF (filgrastim 300 μg/m^2^ or lenograstim 5 μg/kg s.c.) is initiated on Day 1 and continues until the WBC counts reach around 5 x 10^9^/L.

### 3.6 Study procedures-maintenance

**Figure 4:**
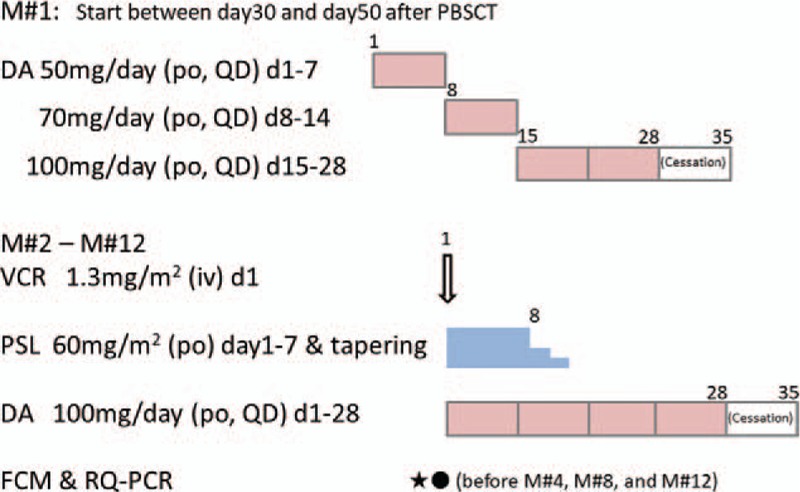
Maintenance regimens. DA = dasatinib, FCM = flow cytometry, PBSCT = peripheral blood stem cell transplantation, PSL = prednisolone, RQ-PCR = real-time quantitative polymerase chain reaction, VCR = vincristine.

Maintenance therapy is started between Day 30 and Day 50 after transplantation when the patient can undoubtedly take oral medication. As maintenance course #1, DA is initiated as a dose of 50 mg/day, and increased up to 100 mg/day: 50 mg/day on Days1 to 7, 70 mg/day on Days 8 to 14, 100 mg/day on Days 15 to 28, and then ceased on Days 29 to 35. As maintenance course #2–12, a combination of VCR, PSL, and DA is administered: VCR 1.3 mg/m^2^ on Day 1, PSL 60 mg/m^2^ on Days 1 to 7, and DA 100 mg/day on Days 1 to 28 every 35 days (Fig. 4).

### 3.7 Minimal residual disease and tumor immunology

In connection with relapse, MRD using RQ-PCR for detection of *BCR-ABL* chimeric gene and T-cell subsets using flow cytometry, especially FOXP3 + CD25 + CD4 + regulatory T cells (Tregs),^[20,21]^ are analyzed at designated time points: before conditioning for transplantation, Day 30 after transplantation, before maintenance courses #4, #8, and #12, and 3 years after transplantation.

## 4 Discussion

This is an exploratory study for the safety and efficacy of auto-PBSCT for Ph + ALL. It is generally recognized that autologous transplantation has a higher risk of relapse than allogeneic transplantation due to lack of allogeneic immunity but a much lower risk of NRM. Higher age, especially 55 years or older, was reported to be a significant risk factor for NRM after allogeneic transplantation for Ph + ALL.^[15]^ To minimize the risk of NRM and relapse, this study targets patients aged 55 years or older with CMR.

The chemomobilization regimen for PBSCH varies depending on studies. ^[22,23]^ It is common for patients with malignant lymphoma or multiple myeloma to receive high-dose CPM or multidrug chemotherapy and G-CSF. For multidrug chemotherapy, there are several reports using a combination of CPM, anthracycline, and steroids,^[24–26]^ which are commonly used in combination for Ph + ALL. Therefore, in this study, we chose the Japan Adult Leukemia Study Group (JALSG) Ph + ALL213 consolidation C2 regimen for PBSCH, which was one of widely used chemotherapy regimens for Ph + ALL in Japan (UMIN000012173).

There is no specific preparative regimen of auto-PBSCT for Ph + ALL. Previous studies have used preparative regimens for allogeneic transplantation, or auto-PBSCT for malignant lymphoma or multiple myeloma.^[6,7]^ In this study, the LEED regimen is used for the following reasons: The regimen is commonly used for auto-PBSCT of malignant lymphoma in participating hospitals; Each drug used in the LEED regimen is covered by insurance in Japan for acute leukemia; and Etoposide has been a key drug of preparative regimen for ALL.^[27–29]^

Tregs have received a lot of attention in relation to cancer immunity in recent years. Tregs suppress antitumor immunity and contribute to tumor progression and metastasis.^[21,30]^ On the contrary, in cancer patients, effector cells including CD8 + T cells are primed and expanded to suppress tumors. Therefore, the balance of Tregs and effector T cells will affect the prognosis. In chronic myeloid leukemia patients, it was reported that the number of Tregs was significantly lower in the CMR group than in the No-CMR group.^[31]^ Our hypothesis is that dominant recovery of effector T cells after auto-PBSCT will contribute to long-term relapse-free survival in Ph + ALL patients.

This study can provide a foundation of auto-PBSCT for Ph + ALL. MRD-based strategies would identify patients who could achieve long-term remission without allo-HCT and lead to safer treatment for Ph + ALL.
